# DOTAP-Based Hybrid Nanostructured Lipid Carriers for CRISPR–Cas9 RNP Delivery Targeting *TGFB1* in Diabetic Nephropathy

**DOI:** 10.3390/pharmaceutics18010094

**Published:** 2026-01-11

**Authors:** Nurul Jummah, Hanifa Syifa Kamila, Satrialdi, Aluicia Anita Artarini, Ebrahim Sadaqa, Anindyajati, Diky Mudhakir

**Affiliations:** 1 Department of Pharmaceutics, School of Pharmacy, Institut Teknologi Bandung (ITB), Bandung 40132, Indonesia; nuruljummah.dty@uim-makassar.ac.id (N.J.); 20725017@mahasiswa.itb.ac.id (H.S.K.); satrialdi@itb.ac.id (S.); 2 Department of Pharmacy, Faculty of Mathematics and Natural Science, Universitas Islam Makassar, Makassar 90245, Indonesia; 3 Biotechnology Laboratory, Department of Pharmaceutics, School of Pharmacy, Institut Teknologi Bandung (ITB), Bandung 40132, Indonesia; anita.artarini@itb.ac.id (A.A.A.); anin@itb.ac.id (A.); 4 Research Center for Vaccine and Drugs, National Research and Innovation Agency (BRIN), Bogor 16911, Indonesia; ebrahim.saad.ebrahim.sadaqa@brin.go.id

**Keywords:** CRISPR–Cas9 RNP, hybrid NLCs, DOTAP, TGF-β1, diabetic nephropathy, gene delivery

## Abstract

**Background**: Diabetic nephropathy (DN) is largely driven by transforming growth factor-β1 (TGF-β1)-mediated fibrosis. Clustered regularly interspaced short palindromic repeats (CRISPR)-associated protein 9 (Cas9) ribonucleoprotein (RNP) complexes offer precise gene disruption, yet effective non-viral delivery remains a challenge. This study developed cationic lipid-based hybrid nanostructured lipid carriers (NLCs) for intracellular delivery of *TGFB1*-targeting RNP as an early-stage platform for DN gene modulation. **Methods**: A single-guide RNA (sgRNA) targeting human *TGFB1* was assembled with Cas9 protein (1:1 and 1:2 molar ratios). Hybrid NLCs comprising squalene, glyceryl trimyristate, and the cationic lipid 1,2-dioleoyl-3-trimethylammonium-propane (DOTAP) were formulated via optimized emulsification–sonication to achieve sub-100 nm particles. Physicochemical properties, including polydispersity index (PDI), were assessed via dynamic light scattering (DLS), while silencing efficacy in HEK293T cells was quantified using quantitative reverse transcription PCR (RT-qPCR) and enzyme-linked immunosorbent assay (ELISA). **Results**: Optimized NLCs achieved hydrodynamic diameters of 65–99 nm (PDI < 0.5) with successful RNP complexation. The 1:2 Cas9:sgRNA formulation produced the strongest gene-editing response, reducing *TGFB1* mRNA by 67% (*p* < 0.01) compared with 39% for the 1:1 ratio. This translated to a significant reduction in TGF-β1 protein (*p* < 0.05) within 24 h. **Conclusions**: DOTAP-based hybrid NLCs enable efficient delivery of CRISPR–Cas9 RNP and achieve significant suppression of *TGFB1* expression at both transcriptional and protein levels. These findings establish a promising non-viral platform for upstream modulation of profibrotic signaling in DN and support further evaluation in kidney-derived cells and in vivo renal models.

## 1. Introduction

Diabetic nephropathy (DN) is a progressive microvascular complication of diabetes mellitus and remains a leading cause of end-stage renal disease worldwide. The condition is characterized by persistent albuminuria, a gradual decline in glomerular filtration rate, accumulation of extracellular matrix, and structural remodeling within the glomerulus and tubulointerstitium [1,2,3]. Although current therapeutic options such as glucose control and renin–angiotensin system inhibition can delay disease progression, they do not address the molecular mechanisms that drive renal fibrosis [4]. Many patients therefore continue to progress toward irreversible renal failure. This ongoing clinical burden highlights the need for therapeutic strategies that target upstream molecular pathways responsible for tissue injury.

Transforming growth factor beta 1 (TGF-β1) has been extensively implicated as a key mediator of renal fibrosis in DN [5]. Hyperglycemia, reactive oxygen species, and advanced glycation end-products stimulate TGF-β1 upregulation in glomerular cells, particularly within the mesangial compartment. Increased TGF-β1 activity enhances extracellular matrix production, promotes mesangial cell proliferation, and contributes to structural remodeling of the glomerulus. Numerous studies have demonstrated that reducing TGF-β1 expression or signaling can mitigate renal fibrosis in experimental models [6,7]. However, strategies based on monoclonal antibodies, siRNA, or miRNA often exhibit limited stability, insufficient cellular penetration, and short duration of action [8,9]. These limitations indicate the need for gene-modulation approaches that achieve more sustained and precise control of *TGFB1* expression.

The Clustered Regularly Interspaced Short Palindromic Repeats CRISPR-associated protein 9 (CRISPR Cas9) genome-editing system provides a powerful platform for targeted gene disruption [10]. The Cas9 ribonucleoprotein (RNP) complex, guided by a single-guide RNA, introduces sequence-specific DNA double-strand breaks that suppress gene expression through endogenous repair mechanisms [11]. Although the technology has transformed gene-based therapeutics, its successful application depends strongly on effective intracellular delivery [12]. Cas9 protein and guide RNA are large, negatively charged, and vulnerable to enzymatic degradation, which makes their delivery into cells a major barrier. A suitable non-viral carrier is therefore essential to protect the RNP complex, facilitate cellular uptake, and support intracellular trafficking toward the nucleus [13]. Recent advances in CRISPR–Cas9 gene-editing technology have opened new therapeutic avenues for genetic disorders such as sickle cell anemia and β-thalassemia and are currently being explored in clinical trials for various diseases, including renal pathologies. However, CRISPR/Cas9-based targeting of long non-coding RNAs (lncRNAs) remains in its early stages. While preclinical studies suggest its potential utility in kidney diseases, clinical translation in acute and chronic kidney disorders is still limited, and significant biological challenges remain [14].

Lipid-based nanocarriers have emerged as promising vehicles for the delivery of nucleic acids and protein therapeutics. Their physicochemical characteristics can be engineered through lipid composition to control particle size, surface charge, stability, and molecular interactions. NLCs, which incorporate both solid and liquid lipid components into a partially ordered matrix, offer enhanced colloidal stability and improved loading capacity [15]. Incorporating a cationic lipid such as 1,2-dioleoyl-3-trimethylammonium propane (DOTAP) introduces positively charged domains that facilitate electrostatic association with the negatively charged Cas9 RNP. Particle size is also an important formulation parameter because it influences colloidal stability, cellular uptake, and intracellular trafficking [16]. For applications that may later extend to renal tissues, nanoscale optimization is particularly relevant; however, overall biological performance ultimately depends on the combined effects of size, charge, lipid composition, and surface characteristics [17]. Despite the therapeutic relevance of TGF-β1 in DN, only a limited number of studies have explored CRISPR Cas9 delivery using custom-engineered lipid systems that could support future renal-targeted applications. This represents a notable gap in current gene-modulation strategies.

In this study, we introduce a DOTAP-based hybrid nanostructured lipid carriers (NLCs) system designed for the delivery of CRISPR Cas9 RNP targeting the *TGFB1* gene. The system was characterized for its nanoscale features and evaluated for its ability to reduce *TGFB1* expression in HEK293T cells. This work provides an initial in vitro proof of concept and represents a preliminary step toward the development of hybrid lipid-based nanocarriers for gene-modulation strategies relevant to diabetic nephropathy.

## 2. Materials and Methods

### 2.1. Materials

Squalene, glyceryl trimyristate, Span 60, and Tween 80 were obtained from Sigma-Aldrich (St. Louis, MO, USA). The cationic lipid 1,2-dioleoyl-3-trimethylammonium-propane (DOTAP) was purchased from Avanti Polar Lipids Inc. (Alabaster, AL, USA). Cell culture reagents, including Dulbecco’s Modified Eagle Medium (DMEM), fetal bovine serum (FBS), penicillin–streptomycin (Pen–Strep), and Trypsin–EDTA, were acquired from Thermo Fisher Scientific (Waltham, MA, USA). All reagents were of analytical-grade purity Human embryonic kidney cells (HEK293T; ATCC CRL-3216) were kindly provided by Prof. Yoshiharu Matsuura (Research Institute for Microbial Diseases, Osaka University, Japan). The plasmid encoding the sgRNA targeting the human *TGFB1* gene (sg-hTGFβ1), cloned into the pUC57 backbone (Catalog No. U4002G), was obtained from GenScript Biotech Corporation (Kallang, Singapore). The construct contains the synthetic sgRNA protospacer sequence designed for human *TGFB1* and includes the T7 promoter for in vitro transcription.

### 2.2. Construction of sg-hTGFβ1 Plasmid

A single-guide RNA targeting the human *TGFB1* gene (sg-hTGFβ1) was synthesized and inserted into a pUC57 plasmid backbone to generate the sg-hTGFβ1_pUC57 construct. To obtain high-quality plasmid for downstream in vitro transcription and CRISPR–Cas9 RNP assembly, the construct was propagated in *Escherichia coli* (*E. coli*) TOP10 using a Transformation Storage Solution (TSS)–assisted heat-shock method based on a previously described protocol [18]. MgCl_2_, 10% (*w*/*v*) polyethylene glycol 6000 (PEG 6000), and 5% (*v*/*v*) dimethyl sulfoxide (DMSO) to enhance cell permeability.

Briefly, a single *E. coli* TOP10 colony was inoculated into Luria–Bertani (LB) medium and cultured overnight at 37 °C with shaking at 150 rpm. The culture was diluted 1:20 (*v*/*v*) into fresh LB medium and incubated until it reached an optical density at 600 nm (OD_600_) of 0.3–0.4 (mid-logarithmic phase). Cells were harvested by centrifugation (3000 rpm, 5 min, 4 °C) and gently resuspended in pre-chilled TSS. For transformation, 200 ng of sg-hTGFβ1_pUC57 plasmid was mixed with 200 μL of competent cells and incubated on ice for 30 min. A heat shock was performed at 42 °C for 90 s, after which cells were immediately returned to ice. Transformants were recovered in antibiotic-free LB medium for 1 h at 37 °C and subsequently plated onto LB agar containing 100 μg/mL ampicillin.

Following incubation at 37 °C for 16–20 h, a single ampicillin-resistant colony was expanded in LB medium supplemented with 100 μg/mL ampicillin (37 °C, 150 rpm, 16–18 h). Plasmid DNA was isolated using the Geneaid Plasmid Miniprep Kit (Geneaid Biotech Ltd., New Taipei, Taiwan). Construct integrity was confirmed by agarose gel electrophoresis (0.8% agarose, 100 V, 30 min) and diagnostic restriction digestion with *Xba*I (37 °C, 4 h). Finally, the sgRNA sequence fidelity was validated via Sanger sequencing using the M13F primer (Apical Scientific, Selangor, Malaysia) and aligned to the reference protospacer design using Clustal Omega 1.2.4-cmake.

### 2.3. In Vitro Transcription and Purification of sgRNA

The sg-hTGFβ1 insert was excised from the pUC57 plasmid via *Xba*I digestion, and the linearized template was purified using the Zymoclean Gel DNA Recovery Kit (Zymo Research, Irvine, CA, USA). In vitro transcription was performed using the MAXIscript T7 Transcription Kit (Thermo Fisher Scientific, Waltham, MA, USA) according to the manufacturer’s instructions. The reaction mixture was subsequently purified using the GeneJET Gel Extraction Kit (Thermo Fisher Scientific, Waltham, MA, USA) to remove residual nucleotides and enzymes. The yield and purity of the transcript were determined spectrophotometrically (A260/A280 ratio), and the purified sgRNA was stored at −80 °C until RNP assembly.

### 2.4. Assembly of CRISPR–Cas9 RNP Complex

Recombinant Cas9 nuclease (Sigma-Aldrich, St. Louis, MO, USA) was reconstituted to a working concentration of 170 ng/μL. To optimize the loading efficiency, purified sg-hTGFβ1 sgRNA was combined with Cas9 protein at molar ratios of 1:1 and 2:1 (sgRNA:Cas9). The mixtures were incubated at room temperature for 30 min to allow efficient formation of the Cas9–sgRNA RNP complexes. The assembled RNP complexes were then kept on ice and used immediately for formulation.

### 2.5. Formulation of DOTAP-Based Hybrid Nanostructured Lipid Carriers (NLCs)

DOTAP-based hybrid NLCs were prepared using a modified emulsification and sonication method adapted from previously described nanoparticle fabrication protocols [19]. The formulation process was rigorously optimized to achieve a target hydrodynamic diameter of 50–100 nm, a critical size range required for efficient passive targeting and cellular uptake in renal tissue. The lipid phase was composed of squalene (liquid lipid), glyceryl trimyristate (solid lipid), Span 60, and the cationic lipid DOTAP, dissolved in ethanol. The aqueous phase consisted of 10 mM sodium citrate buffer supplemented with Tween 80. To minimize interfacial tension and ensure homogeneity, both phases were heated to 65 °C prior to mixing. The aqueous phase was added gradually to the lipid phase under high-shear homogenization (7200 rpm, 2 min) to generate a coarse pre-emulsion.

To reduce droplet size to the nanoscale, the pre-emulsion was subjected to probe sonication using a 750 W ultrasonic processor equipped with a 6 mm titanium probe. Sonication was performed at 45% amplitude in pulsed mode (15 s on/15 s off) to prevent thermal degradation. A systematic optimization study was conducted to establish the ideal process parameters; variables included the surfactant-to-lipid (S:L) ratio, mixing temperature (50 vs. 65 °C), homogenization speed (5000–7200 rpm), and sonication duration (2–10 min). Based on the design criteria (size < 100 nm, PDI < 0.5), the final optimized protocol utilized an S:L ratio of 2.13, a mixing temperature of 65 °C, and a sonication duration of 10 min. Following formulation, the NLCs were cooled to room temperature. Freshly assembled CRISPR–Cas9 RNP complexes were then gently mixed with the blank NLCs dispersion and incubated at 4 °C for 30 min to promote electrostatic association. Two specific formulations were prepared based on the Cas9:sgRNA molar ratio: F1 (1:1) and F2 (1:2). All samples were stored at 2–8 °C prior to physicochemical characterization. Furthermore, a preliminary stability assessment of the blank nanostructured lipid carriers (NLC) was conducted by maintaining the NLC at 4 °C for a duration of seven days. Variations in particle size and its distribution were evaluated daily throughout the storage period.

### 2.6. Physicochemical Characterization of DOTAP-Based Hybrid NLCs

Particle size (hydrodynamic diameter) and polydispersity index (PDI) were determined by dynamic light scattering (DLS) using a Delsa™ Nano C Particle Size Analyzer (Beckman Coulter, Brea, CA, USA) at 25 °C. Samples were diluted in nuclease-free water to minimize multiple scattering. Zeta potential was measured via electrophoretic light scattering using a Zetasizer Pro (Malvern Instruments Ltd., Malvern, UK). Data represent the mean ± standard deviation (SD) of at least three independent batches.

### 2.7. Cell Culture and Transfection

HEK-293T cells were cultured in DMEM supplemented with 10% FBS, 100 U/mL penicillin, and 100 µg/mL streptomycin at 37 °C in a humidified 5% CO_2_ atmosphere. For transfection, cells were seeded at a density of 3.5 × 10^5^ cells/well in 6-well plates and allowed to adhere for 24 h. Cells were then treated with CRISPR–Cas9-loaded NLCs (F1 or F2) or blank NLCs and incubated for an additional 24 h before downstream analysis.

### 2.8. Quantitative Reverse Transcription Polymerase Chain Reaction (qRT-PCR) Analysis

Total RNA was extracted using TRIzol LS reagent (Invitrogen, Carlsbad, CA, USA), and only samples with an A260/A280 ratio greater than 1.8 were used for cDNA synthesis. A total of 500 ng RNA was reverse transcribed using the ReverTra Ace qPCR RT Master Mix with gDNA Remover (Toyobo, Osaka, Japan). qPCR was performed using the SensiFAST SYBR No-ROX Kit (Meridian Bioscience, Cincinnati, OH, USA) on a CFX96 Real-Time PCR System (Bio-Rad, Hercules, CA, USA).

Amplification of *TGFB1* was conducted using the following primers: Forward 5′-CAGCAACAATTCCTGGCGAT-3′; Reverse 5′-AGATAACCACTCTGGCGAGTC-3′. *β-actin* served as the reference gene. Cycling conditions included 95 °C for 2 min, followed by 40 cycles of 95 °C for 5 s, 60 °C for 10 s, and 72 °C for 20 s. Melt-curve analysis (65–95 °C) confirmed amplification specificity. Relative expression was quantified using the Pfaffl method with efficiency corrections [20,21]. All experiments were performed in triplicate. The following equation was developed by M.W. Pfaffl for simple gene expression calculation [20,21]:

$$\mathrm{Ratio}=\frac{{E_{target}}^{∆Ct\;target\;(control-treated)}}{{E_{ref}}^{∆Ct\;ref\;(control-treated)}}$$

$$\mathrm{Gene}\;\mathrm{expression}\;\mathrm{ratio}=\frac{{E_{TGFB1}}^{∆Ct\;TGFB1}}{{E_{b-actin}}^{∆Ct\;b-actin}}$$

### 2.9. Protein Expression Analysis Using Enzyme-Linked Immunosorbent Assay (ELISA)

TGFβ1 protein levels were quantified using a sandwich ELISA procedure adapted from established protocol [22] with minor modifications. HEK-293T cells were seeded into six-well plates at a density of 3.5 × 10^5^ cells per well and incubated for 24 h to allow cell attachment before treatment. Cells were subsequently exposed to fresh medium containing the designated CRISPR–Cas9-loaded NLCs (F1 or F2) or blank NLCs and incubated for an additional 24 h. Post-treatment, the culture medium was removed, and cells were washed twice with ice-cold 1× Tris-buffered saline (TBS). Ice-cold RIPA Lysis and Extraction Buffer was added to each well, and plates were incubated on ice for 30 min with intermittent gentle pipetting to ensure complete cellular lysis. The lysates were collected and clarified by centrifugation at 15,000× *g* for 12 min at 4 °C using a Hermle Z36 HK centrifuge (Hermle Labortechnik, Wehingen, Germany). The resulting supernatants were collected for analysis.

Total protein concentration was quantified using the Bradford assay (Sigma-Aldrich, St. Louis, MO, USA) with bovine serum albumin (BSA) as the calibration standard. TGF-β1 levels in the clarified lysates were then measured using the Human TGF-β1 ELISA Kit (BMS249-4, Thermo Fisher Scientific, Waltham, MA, USA) according to the manufacturer’s protocol. Absorbance was recorded at 450 nm using a Thermo Fisher Scientific microplate reader (Waltham, MA, USA). All measurements were performed in triplicate, and data were normalized to total protein content.

### 2.10. Statistical Analysis

All data are presented as mean ± standard deviation from at least three independent experiments. Statistical comparisons were performed using an unpaired Student’s *t*-test in Minitab21.1.0.0 (Minitab LLC, State College, PA, USA). Differences were considered statistically significant at * *p* < 0.05, ** *p* < 0.01 and *** *p* < 0.001

## 3. Results

### 3.1. Construction and Validation of the sg-hTGFβ1 Plasmid

**Figure 1:**
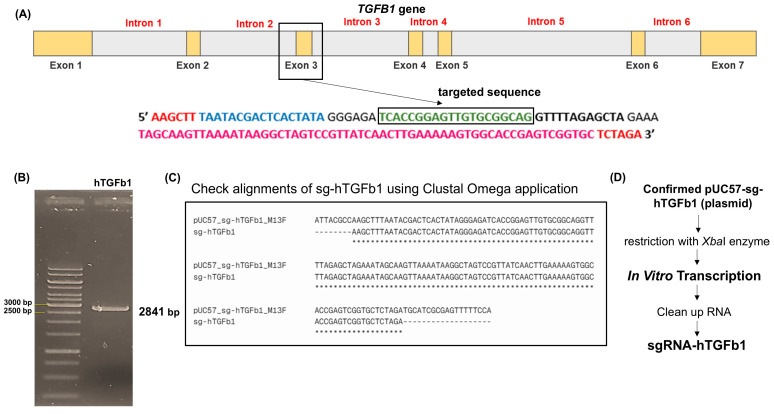
Construction and validation of the sg-hTGFβ1 plasmid and sgRNA preparation. (**A**) Schematic representation of the human TGFB1 gene showing exon–intron structure and the sg-hTGFβ1 target site in exon 3. (**B**) Agarose gel electrophoresis of the sg-hTGFβ1_pUC57 plasmid following *Xba*I digestion, confirming the expected 2841 bp backbone. (**C**) Sequence alignment analysis of sg-hTGFβ1 using the Clustal Omega application. The alignment demonstrates a complete match between the sg-hTGFβ1 sequence and the reference plasmid (pUC57_sg-hTGFβ1_M13F), confirming the fidelity and accuracy of the designed protospacer sequence. (**D**) Workflow outlining sgRNA production, including plasmid confirmation, *Xba*I linearization, in vitro transcription, and purification of the final sgRNA-hTGFβ1 transcript.

The sg-hTGFβ1 construct targeting exon 3 of the human *TGFB1* gene was successfully generated and verified through a series of molecular analyses (Figure 1A). Diagnostic restriction digestion of the sg-hTGFβ1_pUC57 plasmid using *Xba*I yielded the expected fragment corresponding to the pUC57 backbone (2841 bp), confirming correct cloning (Figure 1B). Sanger sequencing further demonstrated complete sequence concordance between the engineered plasmid and the computational sgRNA design, validating the fidelity of the protospacer and scaffold regions (Figure 1C, Figure S1). Following in vitro transcription, the purified sgRNA yielded approximately 5.1 µg (5107.6 ng), providing sufficient material for downstream RNP assembly. This production workflow is summarized in Figure 1D.

### 3.2. Optimization and Physicochemical Characterization of DOTAP-Based Hybrid NLCs

DOTAP-based hybrid NLCs were systematically optimized to achieve an appropriate balance between cationic charge requirements and the stringent size constraints essential for efficient gene delivery. Initial screening of non-cationic lipid matrices yielded small, monodisperse nanoparticles (51.90 ± 5.75 nm, PDI 0.29) at a surfactant-to-lipid (S:L) ratio of 1.85. However, the incorporation of DOTAP—essential for the electrostatic binding of the CRISPR–Cas9 RNP—precipitated a marked increase in hydrodynamic diameter to 127.20 ± 5.95 nm, exceeding the target range for cellular internalization. To counteract this enlargement, the formulation process was refined by applying the specific optimized homogenization and sonication conditions described in Section 2.5. The final optimized protocol established a lipid matrix composition of 3.75% squalene, 0.25% DOTAP, and 0.25% glyceryl trimyristate, stabilized by a surfactant blend yielding an S:L ratio of 2.13. This optimized formulation successfully generated CRISPR–Cas9-loaded NLCs with hydrodynamic diameters ranging from 65 to 99 nm and maintained PDI values < 0.5, confirming a uniform colloidal distribution (Table 1). Zeta potential analysis revealed a shift from near-neutral values for blank NLCs (−0.740 ± 2.130) to slightly negative values for loaded formulations (–2.076 to –2.470 mV). This charge reversal is consistent with the successful surface coordination of the anionic CRISPR–Cas9 RNP cargo, which effectively masks the cationic DOTAP domains, thereby stabilizing the complex.

### 3.3. Validation of Quantitative Assay Specificity and Efficiency

Prior to evaluating the therapeutic efficacy of the CRISPR–Cas9-loaded NLCs, the reliability of the RT-qPCR assay was rigorously validated. Melt-curve analysis demonstrated single, unimodal peaks for both the target *TGFB1* gene (T_m_ = 83.11 ± 0.21 °C) and the reference *β*-actin gene (T_m_ = 86.66 ± 0.23 °C). As shown in Figure S2, the absence of secondary peaks, together with single bands in the inset agarose gel, confirmed specific amplification without primer–dimer formation. Amplification efficiency (E) and linearity (R^2^) were further assessed using serial dilution standard curves (Figure S3). Both primer sets exhibited high linearity (R^2^ ≥ 0.99). Calculated efficiencies were 115% for *β*-actin and 114% for *TGFB1*. To account for these efficiency values and ensure accurate quantification, relative gene expression was calculated using the efficiency-corrected Pfaffl method rather than the conventional 2^−ΔΔC_t_^ approach [20,21].

### 3.4. Optimization of Cas9:sgRNA Ratio for TGFB1 Gene Silencing

**Figure 2:**
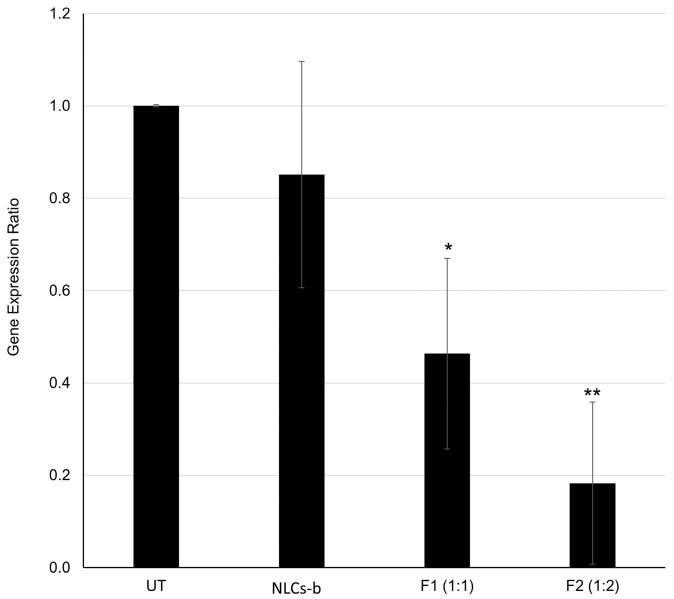
Relative *TGFB1* mRNA expression levels in HEK-293T cells following NLC transfection. Gene expression was quantified 24 h post-treatment using RT-qPCR, normalized to the reference gene *β-actin*, and calculated relative to the untreated control (UT). Experimental groups include blank NLCs (NLCs-b) and CRISPR–Cas9-loaded formulations at Cas9:sgRNA molar ratios of 1:1 (F1) and 1:2 (F2). Data represent the mean ± SD of three independent experiments (*n* = 3). Statistical significance compared to the blank control (NLCs-b) is indicated by * *p* < 0.05 and ** *p* < 0.01.

DOTAP-based hybrid NLCs were evaluated by comparing two Cas9:sgRNA molar ratios: 1:1 (F1) and 1:2 (F2). As shown in Figure 2, cells treated with blank NLCs (NLCs-b) exhibited a gene expression ratio of 0.85 ± 0.25. Although slightly lower than the untreated control (UT), this difference was not statistically significant, confirming the biocompatibility of the carrier system. To evaluate the specific silencing effect of the CRISPR cargo, treatment groups were statistically compared to the blank NLCs. The F1 formulation (1:1 ratio) lowered the expression ratio to 0.46 ± 0.21 (*p* < 0.05 vs. NLCs-b), representing a 39% reduction relative to the vehicle control. Notably, increasing the sgRNA concentration in the F2 formulation (1:2 ratio) significantly enhanced therapeutic efficacy, suppressing the expression ratio to 0.18 ± 0.17 (*p* < 0.01 vs. NLCs-b). This corresponds to a marked 67% reduction in relative expression compared to the blank NLCs, indicating that the 1:2 Cas9:sgRNA stoichiometry provides the optimal formulation for effective gene silencing.

### 3.5. Downregulation of TGF-β1 Protein Expression

To determine whether the transcriptional silencing observed by RT-qPCR translated to a reduction in functional protein, TGF-β1 levels were quantified via ELISA. The reliability of the assay was first confirmed by a standard calibration curve, which demonstrated excellent linearity (y = 0.0016x − 0.1155; R^2^ = 0.9987) over the tested concentration range (Figure S4).

**Figure 3:**
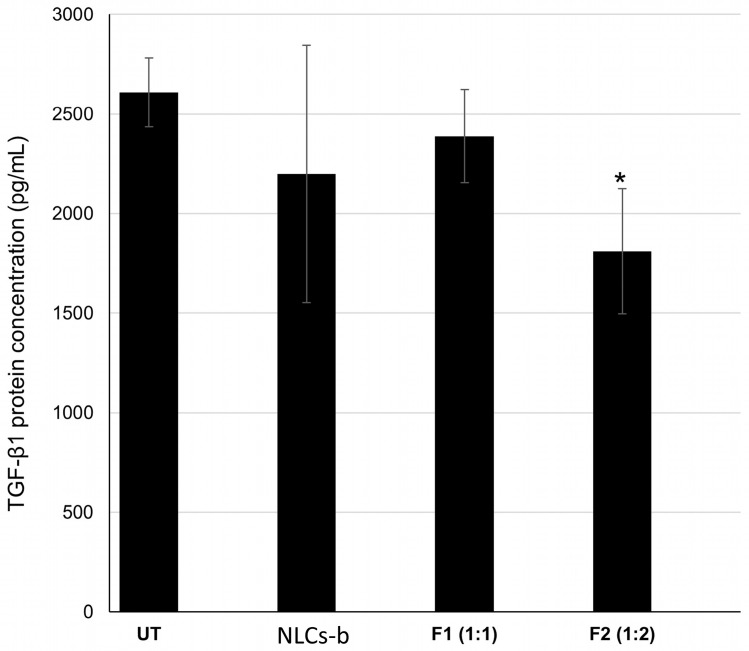
Quantitative analysis of intracellular TGF-β1 protein expression in HEK-293T cells. Protein concentrations (pg/mL) were determined 24 h post-transfection using a sandwich ELISA. Experimental groups include untreated cells (UT), blank NLCs (NLCs-b), and CRISPR–Cas9-loaded formulations at Cas9:sgRNA molar ratios of 1:1 (F1) and 1:2 (F2). Data are presented as mean ± SD from three independent experiments (*n* = 3). Statistical significance relative to the blank formulation (NLCs-b) is indicated by * *p* < 0.05.

As shown in Figure 3, cells treated with blank NLCs (NLCs-b) exhibited protein levels comparable to the UT, confirming that the lipid carrier itself does not significantly alter TGF-β1 production. Among the CRISPR-treated groups, the F1 formulation (1:1 ratio) exhibited a non-significant difference in protein expression compared to the blank control (*p* > 0.05), indicating that this ratio was insufficient to drive translational suppression within the 24 h timeframe. In contrast, the optimized F2 formulation (1:2 ratio) resulted in a significant downregulation of TGF-β1 protein (*p* < 0.05 vs. NLCs-b). The observed 16% reduction in protein levels is more modest than the corresponding mRNA knockdown (67%); however, this discrepancy is expected due to the temporal lag between transcriptional suppression and the natural turnover (half-life) of pre-existing TGF-β1 protein [23]. Collectively, these data demonstrate that the 1:2 Cas9:sgRNA stoichiometry (F2) successfully mediates gene silencing at both the transcriptional and translational levels.

## 4. Discussion

DN progresses through a tightly coordinated network of profibrotic signaling pathways, with TGF-β1 serving as a central upstream regulator of extracellular matrix accumulation, mesangial expansion, and glomerulosclerosis. TGF-β1 promotes epithelial-to-mesenchymal transition (EMT), enhances myofibroblast activation, and suppresses anti-fibrotic signaling pathways, collectively leading to renal scarring and loss of function. Due to its central role in fibrogenesis and disease progression, TGF-β1 is widely recognized as a critical therapeutic target for strategies aimed at attenuating renal fibrosis and preserving kidney function [24]. Given its pivotal role in renal fibrosis, suppression of *TGFB1* has long been considered a promising therapeutic strategy. Yet progress in developing gene-modulating therapies for DN has been constrained by the absence of delivery systems capable of stabilizing, protecting, and transporting large anionic biomacromolecules such as CRISPR–Cas9 RNP complexes. To overcome this limitation, we developed rationally engineered DOTAP-based hybrid NLCs designed to promote stable complexation, protect the RNP cargo, and enable efficient intracellular delivery of Cas9 RNP targeting *TGFB1*, thereby addressing a key translational barrier in DN gene therapy.

The CRISPR–Cas9 RNP format offers several advantages over plasmid or viral systems. RNP delivery allows immediate gene editing upon cell entry, reduces immunogenicity, avoids prolonged nuclease expression, and is generally associated with fewer off-target effects [25,26]. Prior studies targeting *TGFB1* in animal or cancer models relied primarily on lentiviral delivery, which is effective but presents risks of genomic integration and sustained Cas9 expression [27,28]. Delivery of CRISPR as an RNP complex mitigates many of these concerns, but it is highly dependent on an effective carrier because Cas9 protein and sgRNA are susceptible to enzymatic degradation and have limited membrane permeability [29]. A delivery platform that stabilizes the RNP, protects it during extracellular transit, and promotes efficient cellular uptake is therefore essential for enabling therapeutic gene editing in DN.

The mixed-matrix architecture of our hybrid NLCs provides several structural advantages for accommodating and stabilizing large biomacromolecular cargos. The combination of liquid squalene with solid glyceryl trimyristate creates a semi-crystalline lipid matrix with increased defect density, a characteristic that has been reported to enhance loading capacity and reduce crystallinity-driven cargo expulsion [30]. Previous studies using glyceryl trimyristate (Dynasan 114) and squalene have shown that the solid-to-liquid lipid ratio can influence colloidal stability and maintain encapsulation efficiency for nucleic acids [19]. Squalene was added as the core lipid to improve nanoparticle stability, structural integrity, and charge capacity, and its role in improving particle homogeneity has been widely reported. A squalene concentration of 3.75% was selected based on initial optimization, which demonstrated favorable particle size and overall formulation stability (Figure S5). Deviations from this concentration resulted in suboptimal physicochemical properties. Overall, these formulation parameters were selected to produce a stable and efficient NLC system for CRISPR–Cas9 delivery. In line with these findings, our hybrid NLCs structure likely forms a more flexible microenvironment that minimizes aggregation and helps preserve the structural integrity of Cas9 RNP.

The inclusion of DOTAP was essential for electrostatic condensation of the negatively charged RNP. DOTAP is known to improve interaction with cellular membranes and enhance internalization of nucleic acid therapeutics [31]. However, its incorporation initially caused an undesirable increase in particle size. The DOTAP-to-lipid particle ratio was determined through formulation optimization conducted during our research. Several DOTAP ratios were initially evaluated to achieve an appropriate balance between particle stability and surface charge. The final ratio was selected because it provided sufficient positive charge for effective nucleic acid complexation. Through optimization of surfactant composition, homogenization, and sonication parameters, we restored the particle size to the 65–99 nm range. This nanoscale dimension is particularly relevant because particles below 100 nm have been reported to interact more effectively with renal structures, including the glomerular endothelium and mesangial region [32]. Previous studies in renal nanomedicine have shown that nanoparticles within the 6–150 nm range are capable of crossing the glomerular endothelial barrier and that particles between approximately 75 and 95 nm exhibit the most prominent mesangial deposition [33]. This size dependence was demonstrated in an in vivo evaluation of celastrol–albumin nanoparticles, where formulations between 75 and 130 nm were compared. The 75 nm particles achieved the highest mesangial uptake and produced measurable therapeutic benefits in a rat model of mesangioproliferative glomerulonephritis, including reductions in proteinuria, inflammatory activity, glomerular hypercellularity, and extracellular matrix accumulation [34]. These findings support the rationale for selecting a sub-100 nm NLCs formulation and suggest that our optimized size range may be favorable for passive renal exposure and potential mesangial interaction in future in vivo studies. In addition, achieving a near-neutral or mildly negative zeta potential after RNP loading is considered advantageous because nanoparticles with low absolute surface charge typically experience reduced opsonin adsorption and slower clearance by the mononuclear phagocyte system [35]. This behavior may extend systemic circulation time and increase the probability of passive renal exposure, which is particularly relevant for therapies aiming to reach glomerular or mesangial compartments. The combined physicochemical profile of the optimized formulation, including the flexible hybrid matrix, DOTAP-mediated complexation, controlled particle size, and moderated surface charge, provides a strong foundation for its use as a non-viral delivery system for CRISPR-based gene modulation. DOTAP-based hybrid NLCs have been widely used for nucleic acid delivery, including plasmid DNA, siRNA, mRNA, and CRISPR–Cas9 systems. These studies demonstrate the effectiveness of DOTAP-containing lipid nanoparticles in enhancing cellular uptake, endosomal escape, and transfection efficiency across various cell types, supporting the rationale for using DOTAP-based hybrid NLCs in the present work [19,31,36].

The biological performance of the system further underscores the importance of optimizing both the nanocarrier architecture and the stoichiometry of the RNP cargo. A distinct difference in *TGFB1* silencing efficiency was observed between the 1:1 and 1:2 Cas9 to sgRNA ratios, resulting in 39% and 67% transcript reductions, respectively. This observation is consistent with studies demonstrating that an excess of sgRNA enhances formation of active Cas9–sgRNA complexes while reducing the proportion of inactive Apo-Cas9. For instance, Shapiro and colleagues showed that providing sgRNA in molar excess significantly increases genome-editing efficiency in mammalian cells [37]. Pan et al. similarly reported that a 1:2 Cas9 to sgRNA ratio supports near-maximal RNP assembly and editing activity [38]. Current CRISPR gene-editing practices also recommend supplying sgRNA in excess to ensure that most Cas9 molecules are fully loaded and available for target engagement. Taken together, these findings highlight that robust genome editing requires coordinated optimization of both RNP assembly and NLCs design, both of which contributed to the effective *TGFB1* silencing achieved in our system. At the protein level, the optimized formulation produced a measurable reduction in TGF-β1 expression, although the decrease was smaller than the corresponding mRNA knockdown at the 24 h time point. Such temporal divergence is expected because TGF-β1 exhibits slower turnover kinetics, undergoes regulated secretion, and can accumulate in extracellular compartments. Evidence from mammalian and yeast systems similarly shows that reductions in transcript levels do not always translate immediately into proportional decreases in protein abundance, largely due to pre-existing protein pools that persist until they are naturally degraded [23,39]. The early decrease in TGF-β1 protein observed in our study therefore reflects successful intracellular delivery of the RNP and initiation of genomic disruption, and greater reductions would be anticipated at later time points or with repeated administration.

Collectively, these findings position our hybrid NLCs as a promising non-viral platform for modulating profibrotic signaling in DN at its genomic origin. Current therapeutic approaches for DN primarily slow disease progression and do not directly inhibit the molecular pathways that drive fibrosis. CRISPR Cas9 RNP delivery targeting *TGFB1* represents a strategy to intervene upstream of extracellular matrix deposition and has the potential to complement existing renoprotective treatments. Although HEK293T cells offered a controlled environment to confirm intracellular delivery and functional gene modulation, future studies regarding kidney-derived cell lines and relevant animal models will be essential to evaluate renal tropism, biodistribution, durability of gene silencing, and safety. The modular nature of hybrid NLCs also provides opportunities for ligand mediated kidney targeting and for extending the platform to additional fibrotic or inflammatory genes relevant to DN.

## 5. Conclusions

This study successfully engineered a DOTAP-based hybrid NLCs optimized for the stable encapsulation and intracellular delivery of CRISPR–Cas9 RNP complexes targeting the *TGFB1* gene. Through rigorous physicochemical optimization, we achieved a hydrodynamic diameter below 100 nm—a critical parameter for potential passive renal targeting—while maintaining colloidal stability and effective cargo protection. Our biological evaluation identified that a Cas9:sgRNA molar ratio of 1:2 is essential for maximizing therapeutic efficacy, resulting in a robust 67% reduction in *TGFB1* transcripts and a significant suppression of downstream TGF-β1 protein levels in HEK293T cells. These findings collectively validate the compatibility of hybrid NLC architecture with protein–RNA complexes and highlight the importance of RNP stoichiometry in maximizing gene-silencing efficacy. While this work establishes a foundational proof of concept, further studies are needed to evaluate delivery performance in kidney-derived cell lines, assess nanoparticle interactions with renal microstructures, and determine the durability and safety of gene modulation in physiologically relevant models. Nevertheless, the modularity, tunable physicochemical profile, and favorable biological activity of the developed NLCs underscore their potential as a versatile platform for CRISPR-based therapeutic strategies aimed at interrupting profibrotic signaling in diabetic nephropathy and, more broadly, other fibrosis-associated renal disorders.

## Figures and Tables

**Table 1:** Physicochemical characterization of blank and CRISPR–Cas9-loaded DOTAP-based hybrid nanostructured lipid carriers (NLCs). *n* = 3.

Formulation	Particle Size (nm)	Polydispersity Index (PDI)	Zeta Potential (mV)
Blank NLCs	90.00 ± 7.18	0.306 ± 0.025	−0.740 ± 2.130
CRISPR–Cas9 NLCs (1:1)	80.50 ± 15.20	0.281 ± 0.036	−2.076 ± 0.110
CRISPR–Cas9 NLCs (1:2)	89.60 ± 15.80	0.338 ± 0.029	−2.470 ± 0.042

## Data Availability

The raw data supporting the conclusions of this article will be made available by the authors upon request.

## Supplementary Materials

The following supporting information can be downloaded at https://www.mdpi.com/article/10.3390/pharmaceutics18010094/s1, Figure S1: Sanger sequencing chromatograms verifying complete fidelity of the sg-hTGFβ1 protospacer sequence compared with the designed template. Figure S2: Validation of primer specificity via melt curve analysis and agarose gel electrophoresis. Figure S3: Assessment of qPCR amplification efficiency and linearity using standard curves. Figure S4: Standard calibration curve for TGF-β1 ELISA quantification. Figure S5: Evaluation of the short-term stability of blank NLCs stored at 4 °C for 7 days, assessed by (A) particle size and (B) polydispersity index measurements.

## Abbreviations

The following abbreviations are used in this manuscript:

CRISPR	Clustered regularly interspaced short palindromic repeats
DOTAP	1,2-dioleoyl-3-trimethylammonium-propane
NLCs	Nanostructured lipid carriers
PDI	Polydispersity index
mRNA	Messenger RNA
RNP	Ribonucleoprotein
HEK293T	Human embryonic kidney 293T cells
RT-qPCR	Reverse-transcription quantitative polymerase chain reaction
TGF-β1	Transforming growth factor beta 1
TSS	Transformation Storage Solution
sgRNA	Single-guide RNA
ELISA	Enzyme-linked immunosorbent assay
Cas9	CRISPR-associated protein 9
DN	Diabetic nephropathy
DLS	Dynamic light scattering
DMEM	Dulbecco’s Modified Eagle Medium
FBS	Fetal bovine serum
Pen–Strep	Penicillin–streptomycin
