# Supplementary material for: DOTAP-Based Hybrid Nanostructured Lipid Carriers for CRISPR–Cas9 RNP Delivery Targeting TGFB1 in Diabetic Nephropathy
Source: Pharmaceutics. 2026 Jan 11;18(1):94. doi: 10.3390/pharmaceutics18010094 (PMC12844644; doi:10.3390/pharmaceutics18010094)
Supplement: Supplementary file 1 [file pharmaceutics-18-00094-s001.zip › pharmaceutics-4057661-supplementary.pdf]

pharmaceutics

MDPI

Supplementary Materials: DOTAP-Based Hybrid Nanostructured Lipid Carriers for CRISPR–Cas9 RNP Delivery Targeting *TGFB1* in Diabetic Nephropathy

Nurul Jummah, Hanifa Syifa Kamila, Satrialdi, Aluicia Anita Artarini, Ebrahim Sadaqa, Anindyajati and Diky Mudhakir

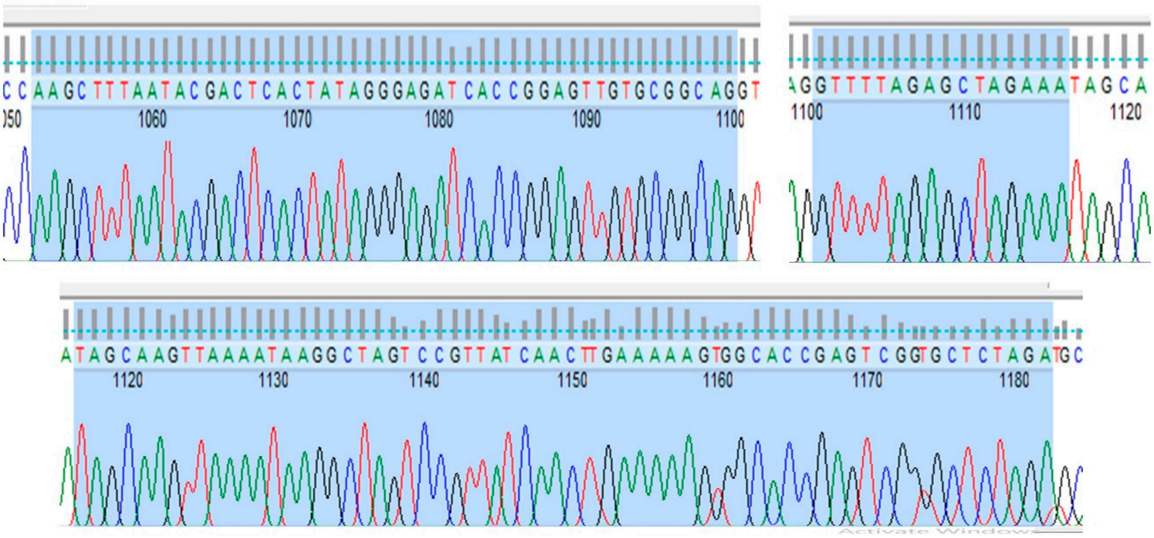

Figure S1. Sanger sequencing chromatograms verifying complete fidelity of the sg-hTGFβ1 protospacer sequence compared with the designed template.

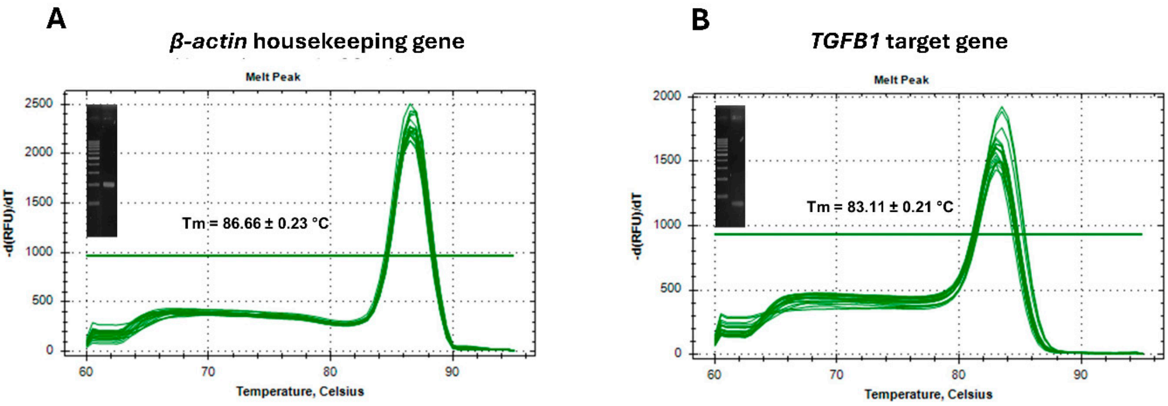

Figure S2. Validation of primer specificity via melt curve analysis and agarose gel electrophoresis for (**A**) the housekeeping *β-actin* and (**B**) the target gene *TGFB1*.

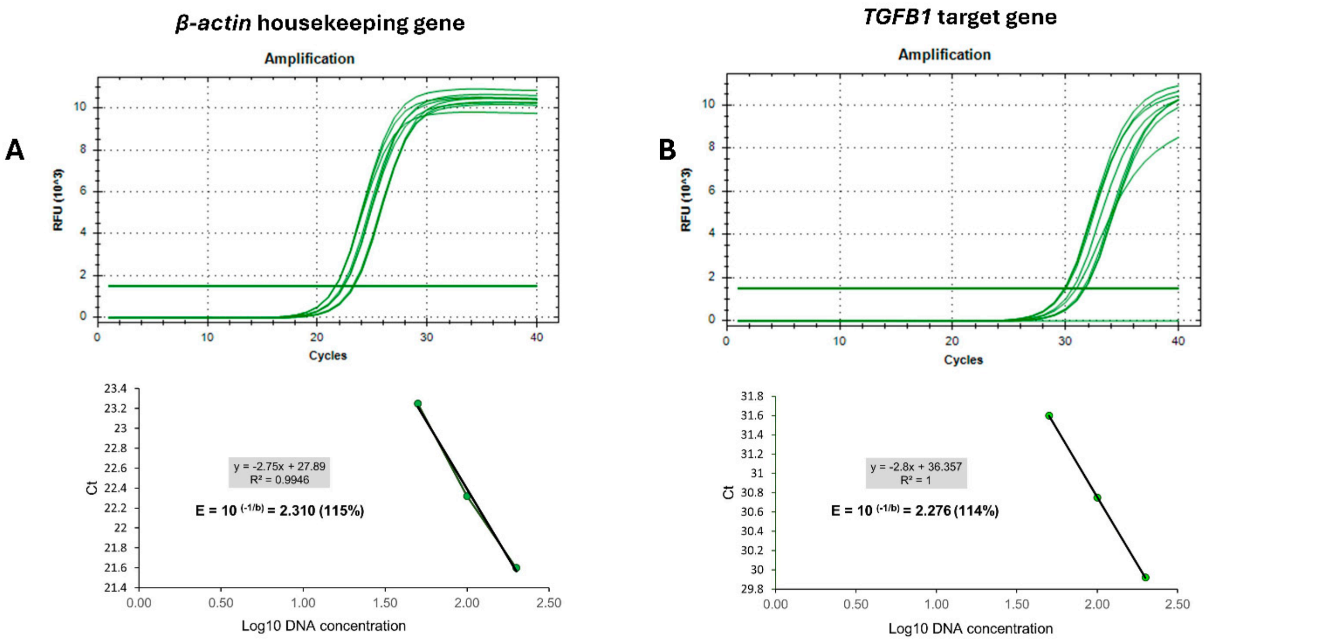

Figure S3. Assessment of qPCR amplification efficiency and linearity using serial dilution standard curves for (**A**) the housekeeping *β-actin* and (**B**) the target gene *TGFB1*, displaying the amplification plots and the corresponding linear regression of Cycle threshold (Ct) versus DNA concentration.

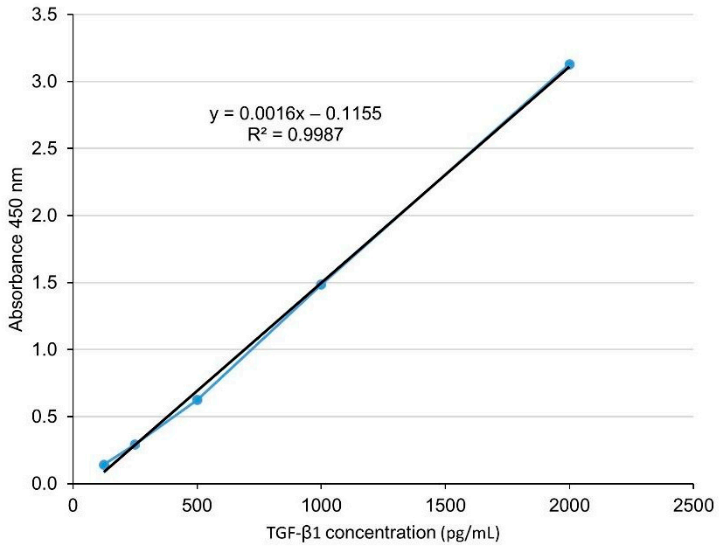

Figure S4. Standard calibration curve for TGF-β1 ELISA quantification demonstrating assay linearity.

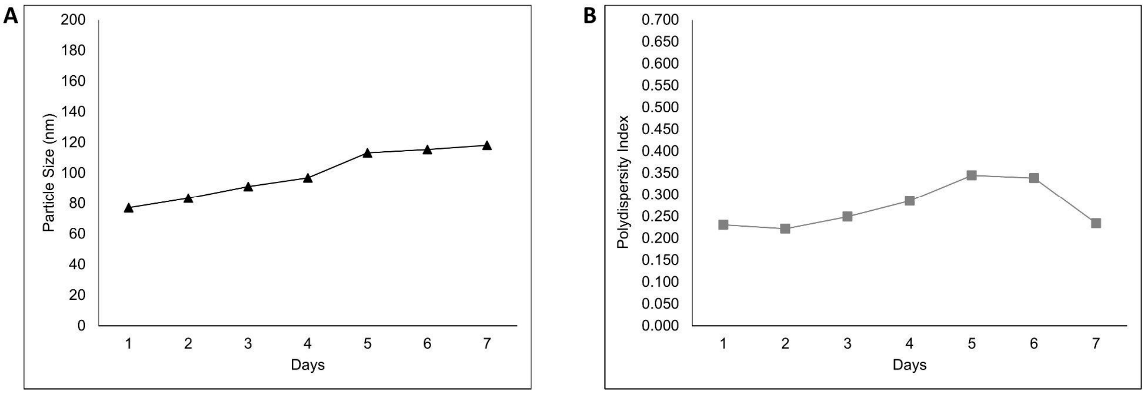

Figure S5. Evaluation of the short-term stability of blank NLCs stored at 4 °C for 7 days, assessed by (**A**) particle size and (**B**) polydispersity index measurements.
